# Type III Secretion 1 Effector Gene Diversity Among *Vibrio* Isolates From Coastal Areas in China

**DOI:** 10.3389/fcimb.2020.00301

**Published:** 2020-06-18

**Authors:** Chao Wu, Zhe Zhao, Yupeng Liu, Xinyuan Zhu, Min Liu, Peng Luo, Yan Shi

**Affiliations:** ^1^Department of Marine Biology, College of Oceanography, Hohai University, Nanjing, China; ^2^Key Laboratory of Marine Bio-Resources Sustainable Utilization, Key Laboratory of Applied Marine Biology of Guangdong Province, South China Sea Institute of Oceanology, Chinese Academy of Sciences, Guangzhou, China

**Keywords:** Vibrios, type III secretion system (T3SS), cytotoxicity, effector, bacterial adhesion

## Abstract

Vibrios, which include more than 120 valid species, are an abundant and diverse group of bacteria in marine and estuarine environments. Some of these bacteria have been recognized as pathogens of both marine animals and humans, and therefore, their virulence mechanisms have attracted increasing attention. The type III secretion system (T3SS) is an important virulence determinant in many gram-negative bacteria, in which this system directly translocates variable effectors into the host cytosol for the manipulation of the cellular responses. In this study, the distribution of the T3SS gene cluster was first examined in 110 *Vibrio* strains of 26 different species, including 98 strains isolated from coastal areas in China. Several T3SS1 genes, but not T3SS2 genes (T3SS2α and T3SS2β), were universally detected in all the strains of four species, *Vibrio parahaemolyticus, Vibrio alginolyticus, Vibrio harveyi*, and *Vibrio campbellii*. The effector coding regions within the T3SS1 gene clusters from the T3SS1-positive strains were further analyzed, revealing that variations in the effectors of *Vibrio* T3SS1 were observed among the four *Vibrio* species, even between different strains in *V. harveyi*, according to their genetic organization. Importantly, Afp17, a potential novel effector that may exert a similar function as the known effector VopS in T3SS1-induced cell death, based on cytotoxicity assay results, was found in the effector coding region of the T3SS1 in some *V. harveyi* and *V. campbellii* strains. Finally, it was revealed that differences in T3SS1-mediated cytotoxicity were dependent not only on the variations in the effectors of *Vibrio* T3SS1 but also on the initial adhesion ability to host cells, which is another prerequisite condition. Altogether, our results contribute to the clarification of the diversity of T3SS1 effectors and a better understanding of the differences in cytotoxicity among *Vibrio* species.

## Introduction

Vibrios are a kind of gram-negative halophilic bacteria widely distributed in marine and estuarine environments (Thompson et al., 2004). To date, more than 120 valid species (https://lpsn.dsmz.de/genus/vibrio) have been identified to be in the genus *Vibrio*, some of which have been recognized as pathogens of marine animals and humans (Austin and Zhang, 2006; Sawabe et al., 2013; Lin et al., 2018). Economic losses and health issues caused by these pathogenic bacteria annually have become common concerns all around the world. Therefore, research on the pathogenic mechanism of these pathogenic *Vibrio* species has received constant and extensive attention, which has led to the identification of some crucial virulence factors, such as thermostable direct hemolysin (TDH) in *V. parahaemolyticus*, cholera toxin (CTX) in *Vibrio cholerae* and cytolysin in *Vibrio vulnificus* (Klose, 2001; Jones and Oliver, 2009; Broberg et al., 2011). Apparently, different *Vibrio* species employ variable virulence strategies, and even different strains of the same species may not rely solely on a given virulence factor.

The type III secretion system (T3SS) is a conserved supermolecular device located on the surface of many gram-negative bacteria (Coburn et al., 2007). Structurally, the T3SS has a remarkable needle-like appearance, which is composed of three major parts: a needle structure extending into the extracellular space, a basal body spanning the inner and outer membranes of the bacteria, and a cytoplasmic sorting platform essential for effector selection and needle assembly (Hu et al., 2014). Structural proteins of the T3SS machinery are highly conserved and evolutionary connected to bacterial flagellum (Hueck, 1998; Diepold and Armitage, 2015). T3SS pathway is capable of secreting a large number of substrates, with the later ones, the T3SS effectors being delivered directly to the host cell cytoplasm (Wagner et al., 2018). In contrast to structural proteins, these effectors vary considerably between different bacterial systems (Troisfontaines and Cornelis, 2005). Elucidation of the molecular roles of various T3SS effectors could provide important insights into the virulence mechanism of different pathogens.

For *Vibrio* spp., the T3SS was first discovered in *V. parahaemolyticus* (serotype O3:K6, strain RIMD2210633) by genome sequencing in 2003 (Makino et al., 2003). The strain has two different sets of T3SS gene clusters, T3SS1 and T3SS2. T3SS1 directly mediates cytotoxicity in host cells during the infection of cultured cells and is present in almost all clinical and environmental isolates of *V. parahaemolyticus*, whereas T3SS2 is typically associated with enterotoxicity during infection in animal models and found to be unique for Kanagawa phenomenon-positive *V. parahaemolyticus* (Park et al., 2004; Hiyoshi et al., 2010). In addition, T3SS gene clusters (T3SS1 or T3SS2) were also found in some other *Vibrio* species, such as *V. alginolyticus, V. harveyi, V. campbellii, Vibrio tubiashii, Vibrio mimicus*, and non-O1/O139 *V. cholerae* (Henke and Bassler, 2004; Park et al., 2004; Dziejman et al., 2005; Okada et al., 2010; Zhao et al., 2010; Liu et al., 2017). Comparative analyses have revealed that most of these clusters are more similar to the T3SS1 of *V. parahaemolyticus* in gene synteny and homology and harbor identical T3SS1 backbones, including all the genes annotated to encode the basal body, the needle-like structure, and the translocator; however, the hypothetical regions (the so-called effector coding regions) located between *vscL* and *vscU* genes of T3SS1, which are supposed to encode the effectors, exhibit many differences in gene content and order among those species (Ono et al., 2006; Zhao et al., 2011; Liu et al., 2017). For example, VopR, an effector encoded by *vp1683* in the *V. parahaemolyticus* strain RIMD2210633, which is involved in the T3SS1-mediated cytotoxicity toward HeLa cells, did not have an ortholog in the T3SS gene cluster of the *V. alginolyticus* strain ZJ51 (Zhao et al., 2010). The T3SS2 was further classified into two clades, T3SS2α and T3SS2β, based on the sequences of the structural genes (Okada et al., 2009). Despite the fact that the T3SS2 was experimentally shown to be functional in only a few *V. cholera and V. parahaemolyticus* strains, effectors in all T3SS2 islands appeared to be pretty variable and performed a wide range of activities (Miller et al., 2019). Moreover, variations in the effectors of T3SS have also been reported in other bacterial systems (Brugirard-Ricaud et al., 2004; Troisfontaines and Cornelis, 2005). Therefore, all these findings prompted us to further characterize the variations in the T3SS effectors among *Vibrio* species and identify the species- or strain-specific effectors.

To this end, we isolated *Vibrio* strains from sea water and marine animals from coastal areas in China and classified those isolates in this study. Subsequently, the distribution of the T3SS gene cluster was examined among the isolated *Vibrio* strains, after which we determined their effector coding regions in the abovementioned T3SS1-postive *Vibrio* strains. Finally, we characterized the variations in the T3SS1 effectors and their ramifications on the cytotoxicity phenotype in fish cells.

## Materials and Methods

### Bacterial Strains and Growth Conditions

Most of the *Vibrio* strains used in this study were isolated from sea water and marine animals collected from coastal areas in South China and the Jiangsu province (Supplemental Table 1). Samples were directly plated onto thiosulfate citrate bile salts sucrose (TCBS, BD, U.S.A) agar without the use of enrichment medium. Inoculated TCBS plates were incubated at 30°C for 12–24 h, and well-isolated colonies were re-streaked onto fresh TCBS for further purification. The monoclonal colony was selected based on colony morphology and stored at −80°C until subsequent use. Some standard *Vibrio* strains (Supplemental Table 1) were purchased from the ATCC (American Type Culture Collection, U.S.A), BCCM/LMG (Belgian Co-ordinated Collections of Micro-organisms, Belgium), or MCCC (Marine Culture Collection of China, China) and used as references. For routine culture, the bacteria were grown in Luria Bertani Broth (LB, BD, U.S.A) supplemented with 2% (w/v) NaCl or in Difco Marine Broth 2216 medium with shaking (200 r.p.m.) at 30°C.

### Common Polymerase Chain Reaction (PCR)

The DNA of all strains in this study was extracted with the MiniBEST Bacteria Genomic DNA Extraction Kit (Takara Bio Inc., Japan). Four conserved genes, the 16S rRNA gene, the glyceraldehyde-3-phosphate dehydrogenase alpha submit (*gapA*) gene, the recombinase alpha subunit (*recA*) gene and the RNA polymerase alpha submit (*rpoA*) gene, were amplified by common PCR using the described primers (Gabriel et al., 2014) and sequenced to identify the isolated *Vibrio* species. The *vcrD, vscC*, and *vopB* genes of the T3SS1, as well as the *vscN2, vscT2*, and *vscR2* genes of the T3SS2 (T3SS2α and T3SS2β, Okada et al., 2010), were examined using their respective primers (Supplemental Table 2) to examine by common PCR whether T3SS1, T3SS2α, and T3SS2β existed in all strains. PCR amplification was conducted using a Premix Ex Taq™ kit (Takara Bio Inc., Japan) following the manufacturer's instructions; the annealing temperature for each gene was adjusted according to the corresponding primers.

### Multilocus Sequence Analysis and Phylogenetic Tree

Multilocus sequence analysis (MLSA) was used to identify the *Vibrio* strains as described previously (Gabriel et al., 2014). Briefly, the gene sequences of the 16S rRNA gene, *gapA, recA*, and *rpoA* were combined to produce concatenated sequence in the order of 16S rRNA gene-*gapA*-*recA*-*rpoA*. The concatenated sequences were aligned using ClustalX (version 1.83), after which a phylogenetic tree was constructed using MEGA 7. A maximum likelihood method was selected, and a bootstrap analysis was employed to quantitatively assess the tree.

### Long Range PCR and Genome Walking

A pair of degenerate primers located at the intersection of the *vscT*/*vscU* and *vscL*/*vscK* genes of the T3SS1, which flank the effector coding regions (Ono et al., 2006), was designed (Supplemental Table 2). Long fragments containing their effector coding regions were amplified from different *Vibrio* strains possessing the T3SS1 using the degenerate primers in long range PCR with LA Taq DNA polymerase (Takara Bio Inc., Japan) as per the manufacturer's recommendations. The sequences were fully determined by using genome walking (Takara Bio Inc., Japan) and subjected to further analysis. Sequence data for the effector coding regions were submitted to the NCBI GenBank and assigned the accession numbers as listed in Supplemental Table 3.

### Identification and Comparison of T3SS1 Effectors in Vibrios

The obtained sequences were compared to the available *Vibrio* genome assemblies (Whole Genome Shortgun, WGS) in NCBI by sequence alignment using BLASTn with the cutoff E-value of 1e-5. A second iteration using BLASTp (E-value 1e-5) were performed to compare the identified putative T3SS1 effectors against non-redundant (nr) database, aiming to find its orthologs in different *Vibrio* species as many as possible. Further, both the type III secretion signals and the binding site of the chaperones (conserved chaperone binding domain, CCBD) for the identified T3SS1 effectors were predicted by EffectiveDB (Eichinger et al., 2015). The putative functional domain modules were classified by PfamScan v1.6 using Pfam32.0 database (Finn et al., 2016). The *Vibrio* genomes that contain the genomic loci spanning the T3SS1 effectors were inspected and compared by MultiGeneBLAST v1.1.14 with default option (Medema et al., 2013).

### Cell Lines and Infection

*Epithelioma papulosum cyprini* (EPC) cells were cultured in M199 medium supplemented with 10% (v/v) fetal bovine serum (FBS, Gibco, U.S.A) at 28°C. For *Vibrio* infection, EPC cells were seeded into 96-well plates and incubated overnight to 90% confluency. Overnight cultures of *Vibrio* isolates were pelleted by centrifugation at 10,000 × g for 2 min at 4°C. The bacterial pellets were resuspended in serum-free M199 medium, and the bacterial suspensions were added to the cell monolayer at a multiplicity of infection (MOI) of 10.

### Lactate Dehydrogenase (LDH) Release Assay

This assay was performed as described previously (Zhao et al., 2018). Briefly, the growth medium of EPC cells in 96-well plates was replaced with 120 μl (per well) of serum-free M199 medium before infection, and the cells were infected with different *Vibrio* strains. At the indicated time point, the 96-well plates were centrifuged at 3,200 × g for 2 min, and 80 μL aliquots of the supernatants were transferred to a new 96-well plate for measuring LDH release by using the Cytotoxicity Detection Kit ^PLUS^ per the manufacturer's instructions (Roche, Switzerland). The background value was measured from the M199 medium only. Maximum LDH release was obtained by total cell lysis using the lysis buffer provided in the kit. Minimum LDH release was obtained from the cell supernatant without infection. Absorbance values from each well were measured at 492 nm by using a microplate spectrophotometer (Spark, Tecan, Switzerland). The results are expressed as a percentage of total cell lysis after subtracting the absorbance value of the background control.

### Bacterial Adhesion Assay

For the adhesion assay, EPC cells were seeded into 24-well plates using M199 medium supplemented with 10% (v/v) FBS and incubated overnight to 90% confluency. Overnight cultures of *Vibrio* strains were pelleted by centrifugation at 10,000 × g for 2 min at 4°C. The bacterial pellets were resuspended in serum-free M199 medium and then adjusted to a bacterial concentration of 5 × 10^7^ CFU/mL. Bacterial suspensions (20 μL) were added to the cell monolayer and cultured at 30°C for 60 min. The cells were first washed with sterile PBS (3 times) to remove non-adherent bacteria, after which the surface-attached bacteria were fully resuspended in bacterial culture medium. Bacteria suspensions were diluted in a 10-fold dilution series, and 20 μL of each diluted concentration was plated onto LB agar supplemented with 2% NaCl. The *Vibrio* plates were cultured at 30°C for an appropriate length of time, and the colonies were finally enumerated to determine the number of adherent bacteria.

### Statistical Analysis

Statistical analysis was performed with a one-way analysis of variance (ANOVA), and *P* < 0.05 were considered statistically significant. Pairwise comparisons were conducted using a Tukey's multiple comparison test, and the analysis was conducted using the SPSS software (version 22.0).

## Results

### Identification of *Vibrio* Isolates

In total, 110 *Vibrio* strains were used in this study, including 98 strains isolated from the coastal areas of South China and the Jiangsu Province (Supplemental Table 1). The MLSA method was utilized to identify these strains. To assess the accuracy of the identification, 12 known *Vibrio* strains belonging to 10 different species (Supplemental Table 1) were included and randomly put together in our MLSA. Phylogenetic trees constructed using maximum likelihood showed that the 12 known *Vibrio* strains were classified into the right clades as expected (Supplemental Figure 1), and the 98 *Vibrio* strains we isolated were sorted into 19 different *Vibrio* species (Supplemental Table 1). Among these 19 species, *V. alginolyticus, V. parahaemolyticus* and *V. harveyi* were the top three most abundant *Vibrio* species, with 23, 20, and 10 isolates, respectively.

### Distribution of the T3SS-Related Genes Among the Examined *Vibrio* Species

To determine the presence of the T3SS genes (T3SS1 and T3SS2) in our *Vibrio* collection, we designed PCR primers targeted to three T3SS1 genes [for the apparatus (VcrD and VscC) and translocon (VopB)] and selected six PCR primer pairs that were used in a previous study for targeting several T3SS2α and T3SS2β genes (*vscN*2, *vscT2*, and *vscR*2). Screening of all *Vibrio* strains revealed that 62 strains exhibited positive signals for the three T3SS1 genes (Supplemental Table 1), all of which represented members of *V. parahaemolyticus, V. alginolyticus, V. harveyi*, and *V. campbellii*. However, the detection of the T3SS2α and T3SS2β genes showed negative signals for almost all *Vibrio* strains, except for *V. parahaemolyticus* strain RIMD2210633 and strain ATCC33847. These two strains were the only T3SS2α-positive strains. These data suggested that the T3SS1, but not the T3SS2, is widely present in all strains of *V. parahaemolyticus, V. alginolyticus, V. harveyi*, and *V. campbellii* in coastal environments in China.

### Genetic Organization of Effector Coding Regions of the T3SS1 in the T3SS1-Positive Strains

To further study variation of the T3SS1 effectors among all T3SS-postive *Vibrio* strains, we determined the nucleotide sequences of the non-conserved regions (here referred to as effector coding regions), which are located between the *vscL* and *vscU* genes of the T3SS1. Sequence analysis showed that the length of their effector coding regions varied obviously among the different *Vibrio* strains, ranging from ~6.9 to 11.7 Kb. However, the GC content of those sequences only ranged from 43 to 47%, which was also similar to the average GC percentage of the genome sequence of Vibrios. A phylogenetic study was undertaken based on the nucleotide sequences of effector coding regions. The resulting tree showed that these *Vibrio* strains were divided into the four distinct groups: *V. parahaemolyticus, V. alginolyticus, V. harveyi*, and *V. campbellii* (Supplemental Figure 2), and exhibited similar taxonomic status in the harveyi clade of the MLSA tree. These findings suggest that acquisition of the T3SS1-related genes may occur in an ancient evolutionary event.

**Figure 1:**
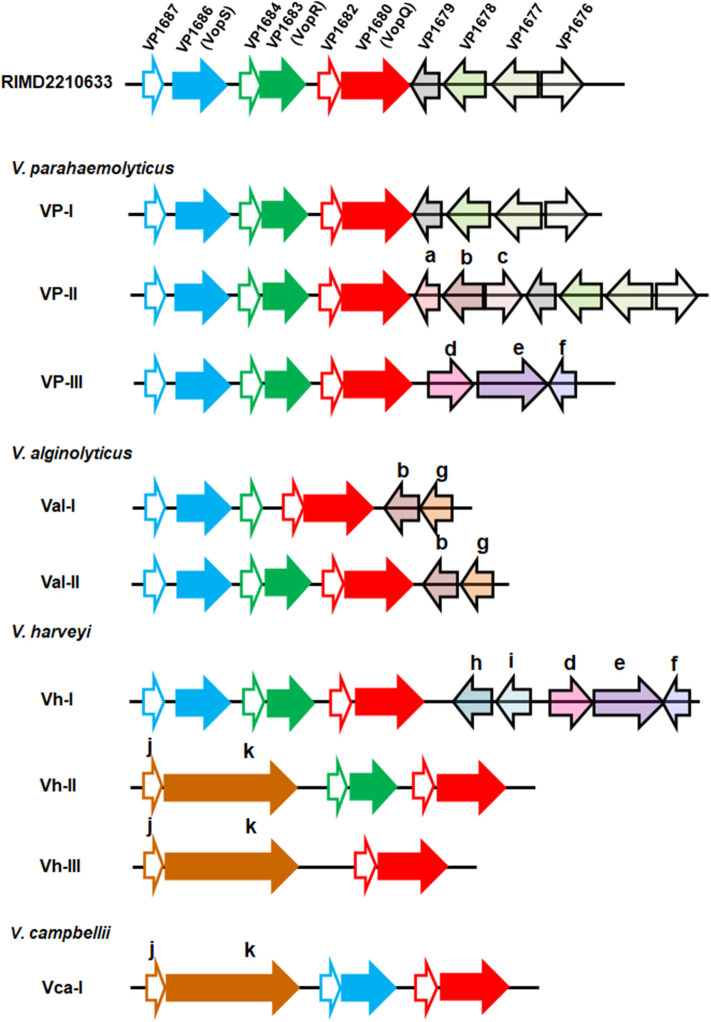
Genetic organization of the T3SS1 effector coding regions in four *Vibrio* species. Each gene was represented by an arrow indicating the approximate size and the direction of transcription based on the position of methionine initiation and the termination codons. The effector coding region of T3SS1 in the *V. parahaemolyticus* strain RIMD2210633 was set as a reference, and different genes within this region were highlighted with different colors. The one-to-one relationship between each indicated effector protein and its adjacent chaperone were shown by identical color code, and the latter was filled with white color. Nine types of genomic organization were classified based on gene content and the order of the T3SS1 effector coding regions from 62 *Vibrio* strains across the four species (*V. parahaemolyticus, V. alginolyticus, V. harveyi*, and *V. campbellii*). The gene (arrow) in the nine types was marked with the same color if it had an ortholog in the reference strain RIMD2210633 or with another color and indicated with a lower case letter as follows: a: GNAT family N-acetyltransferase; b: EamA family transporter, c: LysR family transcriptional regulator; d: HlyD family secretion protein; e: MFS transporter; f: TetR/AcrR family transcriptional regulator; g: AraC family transcriptional regulator; h: SDR family oxidoreductase; i: MarR family transcriptional regulator; j: class I chaperone; k: Afp17.

These sequences were further annotated, and their genetic organizations were compared. Genomic organization of the T3SS1 effector coding region from the *V. parahaemolyticus* strain RIMD2210633, the most studied *Vibrio* T3SS1 by far, was set as a reference, and three known effectors, VP1686 (VopS), VP1683 (VopR), and VP1680 (VopQ), were the primary focus for the comparison analysis. According to gene content and synteny, the organizations of the T3SS1 effector coding regions of 61 strains (except RIMD2210633) were classified into nine different types (Figure 1). As expected, certain variations within the effector coding regions among the four *Vibrio* species, even between different strains of the same species were found. As for the three known effectors, their counterparts were found to be present in almost all *V. parahaemolyticus* and *V. alginolyticus* strains, except for two *V. alginolyticus* strains (E401 and A056) belonging to the type Val-I lacking the homologous protein of VopR. However, *V. harveyi* strains exhibited greater variations in their effectors in this region. The type Vh-I harbored all three known effectors, while the types Vh-II and Vh-III only had two (VopQ and VopR) and one (VopQ) of these effectors, respectively. In other words, the latter two types of *V. harveyi* did not harbor the VopS homolog. Interestingly, they both contained *afp17*, a large gene predicted to encode a protein (Afp17) with an ADP-ribosyltransferase domain in its C-terminal according to the PfamScan results. In addition, strong T3SS secretion signals were also found in the amino terminal of Afp17 on the basis of EffectiveDB prediction (Supplemental Table 4). For the six *V. campbellii* strains, only one type of gene organization of the T3SS effector coding region was found; these strains not only had homologs of VopS and VopQ but also harbored Afp17. Interestingly, a small gene was found to be located adjacently upstream of *afp17* in certain strains of *V. harveyi* and *V. campbellii* from the present study. Importantly, similar structural linkage between these two genes were also found in several other *Vibrio* species, such as *Vibrio owensii, Vibrio tubiashii, Vibrio europaeus, Vibrio chagasii*, and *Vibrio lentus*, as evidenced by gene cluster comparisons (Supplemental Figure 3). The protein encoded by such small gene was considered to be a molecular chaperone for Afp17 since the correct translocation of effectors secreted via the T3SS requires their cognate chaperones for their own stabilization (Akeda and Galan, 2005). Similarly, there were homologs of VP1687, VP1684, and VP1682, serving as the chaperones for VopS, VopR, and VopQ, respectively, in close proximity to their effectors. Four unknown genes in the T3SS1 of the reference strain, *VP1679, VP1678, VP1677*, and *VP1676*, were also compared, revealing that their homologous genes were only present in *V. parahaemolyticus* VP-I and VP-II. However, some other genes that were annotated to be transcriptional regulators (Lys family, AcrR family, AraC family, and MarR family), transporters (EamA, MFS, and HlyD) and enzymes (N-acetyltransferase, oxidoreductase) were also found in the different types.

### Cytotoxicity of Different *Vibrio* Isolates Toward Fish Cells

**Figure 2:**
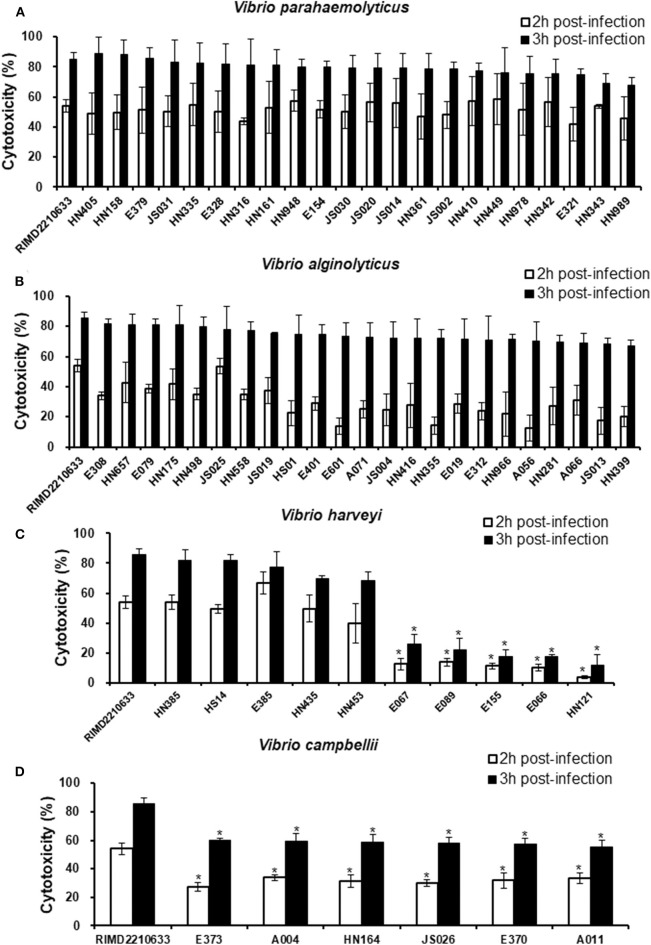
Cytotoxicity toward fish cells induced by different *Vibrio* strains. EPC cells were infected with different strains from *V. parahaemolyticus* **(A)**, *V. alginolyticus* **(B)**, *V. harveyi* **(C)**, and *V. campbellii* **(D)** as described in the Materials and Methods. At the indicated time points, the culture supernatants were measured for the release of LDH, followed by the calculation of cytotoxicity as a percentage of total cellular lysis. The data are expressed as the means ± SE from three independent experiments (*n* = 3). *P < 0.05 by one-way ANOVA and Tukey's multiple comparison test.

Considering the variation in the effectors of the T3SS among the different species, especially in *V. harveyi* strains, we measured the cytotoxicity toward fish cells of all *Vibrio* strains that harbored the T3SS and assessed the correlation between the variation and cytotoxicity. The LDH release assays showed that all *V. parahaemolyticus* strains exhibited a similar cytotoxicity as the reference strain RIMD2210633 toward EPC cells, as evidenced by ~60 and 80% total LDH release at 2 and 3 h post-infection, respectively (Figure 2A). Similarly, the LDH levels in the medium of cells infected with all of the *V. alginolyticus* strains were not significantly different from that of cells infected with the reference strain RIMD2210633 (Figure 2B). For *V. harveyi* strains, the difference in cytotoxicity effects was quite obvious among these strains (Figure 2C). The five strains (HN385, HN435, HN453, E385, and HS14) of *V. harveyi* with the *Afp17* produced equivalent LDH release levels on fish cells when compared to the reference strain RIMD2210633, whereas another five strains (E066, E067, E089, E155, and HN121) caused the lowest cytotoxicity effect, with ~20% total LDH release at 3 h post-infection. Additionally, six *V. campbellii* strains showed a moderate cytotoxicity effect when compared to the reference strain and the *V. harveyi* strains.

### Adhesion Ability Affects T3SS-Mediated Cytotoxicity

**Figure 3:**
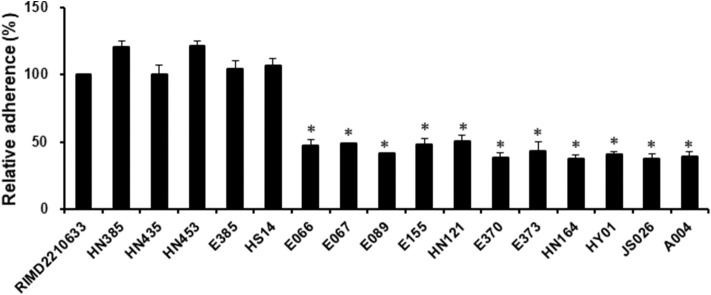
Assay to test adhesion of the *V. harveyi* and *V. campbellii* strains to fish cells. EPC monolayers were infected with all *V. harveyi* and *V. campbellii* strains as described in the Materials and Methods. The *V. parahaemolyticus* strain RIMD2210633 was included as a control. After 1 h of infection, the adherent bacteria were enumerated. The y-axis represented the adhesion efficiency of each strain indicated in the x-axis, relative to strain RIMD2210633. The data are expressed as the means ± SE from three independent experiments (*n* = 3). *P < 0.05 by one-way ANOVA and Tukey's multiple comparison test.

Adherence is a critical first step in the establishment of infection, which then affects the subsequent events, including bacterial pathogenesis (Kline et al., 2009). Since some strains of *V. harveyi* were shown to cause a lower cytotoxicity than the reference strain RIMD2210633, even though they possessed the same effector repertoires (VopS, VopR, and VopQ), it was assumed that differences may exist in the adhesion ability between these strains. For this reason, seventeen strains, as indicated in Figure 3, were selected to measure their adhesion ability. The data showed that the *V. harveyi* strains with a low cytotoxicity effect appeared to have a weaker adhesion ability than the *V. harveyi* strains with high cytotoxicity and the reference strain RIMD2210633 at 1 h post-infection (Figures 2, 3). Furthermore, all *V. campbellii* strains also exhibited low adhesion and consequently did not produce cytotoxicity levels as high as expected, even if they carried the three key effector genes (Afp17, VopS, and VopQ) in their T3SS1 gene cluster. We therefore surmised that the initial adhesion to host cells is a prerequisite factor that affects T3SS-mediated cytotoxicity in *Vibrio* species.

## Discussion

*Vibrio* is a genetically and metabolically diverse group of bacteria that often predominate in the aquatic environment and accounts for more than 10% of the culturable bacterial community (Yooseph et al., 2010; Gilbert et al., 2012; Takemura et al., 2014). In this study, we obtained 19 different *Vibrio* species from the coastal areas of South China and the Jiangsu province, and the most abundant species were found to be *V. parahaemolyticus, V. alginolyticus*, and *V. harveyi*. This was in agreement with the previous reports indicating that the members of the harveyi clade are usually the dominant groups in Chinese coastal environments (Xie et al., 2005; Austin and Zhang, 2006; Chen et al., 2011; Han et al., 2015; Zuo et al., 2019).

The T3SS genes are commonly used as virulence markers for pathogenic bacteria. The T3SS in the *Vibrio* species was discovered from a *V. parahaemolyticus* clinical strain that carried the T3SS1 and T3SS2 gene clusters (Makino et al., 2003). In addition to *V. parahaemolyticus*, the T3SS1 and T3SS2 gene clusters were also found to be present in many other *Vibrio* species. Of these, the T3SS1 gene cluster was mainly present in several species of the harveyi clade, such as *V. alginolyticus, V. harveyi*, and *V. campbellii* (Henke and Bassler, 2004; Park et al., 2004; Zhao et al., 2010; Liu et al., 2017). This conclusion was well-supported by our data showing that all tested strains of *V. parahaemolyticus, V. alginolyticus, V. harveyi*, and *V. campbellii* in our collections possessed the T3SS1 gene cluster. T3SS2-related gene cluster is located within an 80 kb pathogenicity island (Vp-PAI) and the T3SS2 has two distinct subtypes, T3SS2α and T3SS2β (Noriea et al., 2010), and has been reported in some specific strains of non-O1/O139 *V. cholerae*, non-toxigenic O1 *V. cholerae* and *V. mimicus* (Dziejman et al., 2005; Okada et al., 2010; Mahmud et al., 2014). However, six genes within the T3SS2α and T3SS2β were not detected in our isolates, including all the *V. parahaemolyticus* strains. This may have been possible because our *Vibrio* isolates belonged to environmental strains (isolated from sea water or common marine animals), while the T3SS2 genes tend to be closely linked with clinical strains (Okada et al., 2010).

Effector diversity was commonly observed in many T3SS from several different bacterial taxa (Troisfontaines and Cornelis, 2005). The three secreted effectors (VopS, VopR and VopQ) were identified within the T3SS1 effector coding region between *vscL* and *vscU* genes from *V. parahaemolyticus* (Ono et al., 2006). These effectors play a central role in T3SS1-mediated cell death, with a temporal regulation of events, including autophagy, cell rounding and ultimately cell lysis, in HeLa cells (Burdette et al., 2009; Yarbrough et al., 2009; Salomon et al., 2013). Therefore, the specific regions from all the T3SS1-positive strains were highlighted to characterize the variations in the effectors. The orthologs of VopQ were highly conserved in all strains across four different *Vibrio* species and was referred to here as a core effector. This effector was not only responsible for *V. parahaemolyticus* T3SS1-induced autophagy in HeLa cells (Burdette et al., 2009) but also contributed to T3SS1-induced LDH release in HeLa cells and fish cells (Burdette et al., 2009; Zhao et al., 2018). Another core effector, VopS, was of equal importance for T3SS1-induced cell death and was required for the T3SS1-induced cell rounding phenotype in *V. parahaemolyticus* (Yarbrough et al., 2009). The *V. alginolyticus* ortholog (Val1686) of effector VopS was necessary and sufficient to cause cell rounding and apoptosis (Zhao et al., 2018). It was therefore not surprising that the VopS orthologs were found to be present in all strains of *V. parahaemolyticus, V. alginolyticus* and *V. campbellii*. The exception was in certain *V. harveyi* strains that lacked the VopS homolog in their effector coding regions. Surprisingly, a novel T3SS effector protein (Afp17) containing an ADP-ribosyltransferase domain, was discovered in the strains lacking the VopS homolog. The bacterial proteins carrying this domain are usually toxins, known as ADP-ribosylating toxins, that covalently transfer the ADP-ribose portion of NAD to host proteins and result in a variety of cytotoxic effects (Deng and Barbieri, 2008); for example, many T3SS effectors, including AexT of *Aeromonas salmonicida*, ExoS, and ExoT of *Pseudomonas aeruginosa* and SpvB of *Salmonella* spp., have been verified for their toxin activities (Kaufman et al., 2000; Krall et al., 2000; Burr et al., 2003; Cheng and Wiedmann, 2019). In addition, a strong type III secretion signal was found in the N-terminal of almost all under-investigated Afp17 from several different *Vibrio* species (Supplemental Table 4). Notably, the only exception was found in *V. harveyi* strain HENC-02, of which the T3SS signal for Afp17 was determined to be very low (0.00044). Further studies concerning the prediction of type III secretion signal in Vibrios as well as functional comparisons among these Afp17 orthologs will contribute to an in-depth understanding of the evolution and functional implication of T3SS effector signal. Moreover, current knowledge about the T3SS effectors has suggested that certain signal motif in its N-terminal, which served as binding sites for chaperone proteins to facilitate effectors secretion, can be identified by using machine learning approach (Samudrala et al., 2009; McDermott et al., 2011). However, such CCBD was not found in any of the under-investigated Afp17 orthologs, which possibly could be explained by the lacking of *Vibrio*-originated sequences in the training dataset that had been used in CCBD prediction (Costa et al., 2012; Eichinger et al., 2015). Whilst, considering the existence of structural linkage between afp17 and its upstream chaperone gene in several Vibrios other than *V. harveyi* and *V. campbellii*, it's highly likely that the Afp17 could be a *bona fide* effector within the T3SS1 gene cluster that may exert functions similar to VopS during infection. The effector VopR also existed in the majority of the strains but was not found to be in some strains from the *V. alginolyticus, V. harveyi*, and *V. campbellii* species. It is tempting to speculate that VopR may not be indispensable for T3SS1-induced cell death since the *V. alginolyticus* strain ZJ51 lacking the VopR homolog still exerts a comparative cytotoxic effect with *V. parahaemolyticus* T3SS1 on HeLa cells (Zhou et al., 2009; Zhao et al., 2010, 2011), although VopR was suggested to contribute to cell rounding (Salomon et al., 2013).

Many additional genes were frequently found in the vicinity of effector VopQ in many *Vibrio* strains. Although these genes were located within the effector coding regions of the T3SS1 gene cluster, their products, such as transcriptional regulators, transporters and enzymes, did not appear to be related to the T3SS effectors, according to gene functional annotation and the T3SEdb (Tay et al., 2010). We also predicted the type III secretion signals for their products using EffectiveDB, but no T3SS secretion signal was found in their amino terminus. Furthermore, we also had deleted a gene encoding AraC family transcriptional regulator in T3SS1 of *V. alginolyticus* strain ZJ51, however, the deletion has no any effect on T3SS1-mediated cytotoxicity toward fish cells (data not shown). Sequence analysis showed that those “non-related” genes have the similar GC contents to the effector genes in the effector coding region. Therefore, their presence increased gene diversity within the region encoding the T3SS1 effectors, and a functional correlation with T3SS1 remains elusive.

T3SS function is essentially dependent on its effectors. We therefore compared the cytotoxicity toward cultured fish cell lines of all *Vibrio* strains harboring T3SS1. As expected, all strains of *V. parahaemolyticus* and *V. alginolyticus* exhibited cytotoxicity effects similar to the reference strain RIMD2210633 since they all had homologs of two core effectors, VopQ and VopS. Additionally, five *V. harveyi* strains carrying VopQ and Afp17 also produced equal cytotoxicity, although these *V. harveyi* strains lacked VopS. The data further supported the hypothesis that Afp17 is a novel T3SS1 effector and can substitute for VopS, and experimental evidence for this is currently being generated. Surprisingly, another five *V. harveyi* strains harboring the VopQ and VopS orthologs demonstrated quite low cytotoxicities. This phenomenon was explained by bacterial adhesion ability since *V. harveyi* strains harboring the VopQ and VopS orthologs exhibited a significant reduction in adhesion compared to the reference strain RIMD2210633 and other *V. harveyi* strains. It is reasonable that the translocation of effectors into the host cell cytoplasm by T3SS requires direct contact between pathogen and host (Krachler and Orth, 2011; Erwin et al., 2012). We concluded that the initial adhesion to host cells was a prerequisite factor that affected the cytotoxicity effect of *Vibrio* T3SS1. This point was further supported by cytotoxicity and adhesion assays of *V. campbellii* strains. Our *V. campbellii* strains not only had the two core effectors but also carried the Apf17, and it was therefore expected that they possessed a higher cytotoxicity than other *Vibrio* strains. However, due to their weak adhesion, their cytotoxicity effect was lower than that of the reference strain RIMD2210633. Bacterial adhesion is a complicated process of interaction between a pathogen and its host, and requires adhesive molecules on their surfaces. For Vibrio species, many proteins, such as MAM7, VpadF, and flagellar assembly-associated proteins (flrA, flrB, and flrC), have been reported to be linked to bacterial adhesion (Krachler and Orth, 2011; Liu and Chen, 2015; Luo et al., 2016); however, whether the T3SS1 pathway is involved into the regulation of bacterial adhesion remained to be elucidated.

In summary, we here examined the distribution of the T3SS gene cluster from 110 *Vibrio* strains of 26 different species, including 98 strains isolated from Chinese coastal areas, and found that the T3SS1 gene cluster, but not the T3SS2 (T3SS2α and T3SS2β) gene cluster, was extensively present in our isolated *V. parahaemolyticus, V. alginolyticus, V. harveyi*, and *V. campbellii* strains. We further examined their T3SS1 effector coding regions, revealing that the T3SS1 effectors varied among not only the four different *Vibrio* species, but also the different strains of *V. harveyi*. Importantly, we discovered a potential novel effector, Afp17, in the T3SS1 effector coding region of some *V. harveyi* and *V. campbellii* strains, which may substitute for the core effector VopS and perform a similar function in inducing cytotoxicity. Moreover, we also shown that the cytotoxicity effect of T3SS1 was dependent not only on their effectors but also on the initial bacterial adhesion to host cells.

## Data Availability Statement

The datasets generated for this study can be found in the GenBank.

## Author Contributions

CW, YL, XZ, and ML carried out the experiments. CW, YS, and ZZ designed the experiments and analyzed the data. PL contributed the *Vibrio* strains. CW and ZZ wrote the manuscript. All authors have read and agreed to the published version of the manuscript.

## Conflict of Interest

The authors declare that the research was conducted in the absence of any commercial or financial relationships that could be construed as a potential conflict of interest.

## Supplementary Material

Supplemental Figure 1 Phylogenetic tree of different *Vibrio* strains. The phylogenetic analysis was based on multiple sequences alignment of 164 concatenated sequences of 16S rRNA gene-*gapA*-*recA*-*rpoA* and conducted by MEGA7 using the Kimura 2-parameter model with the maximum-likelihood method, and then the tree was further edited using the Evolview online. Values of bootstrap after 1,000 replications were shown in each node branch represented by the sized circles. 14 *Vibrio* clades defined in the previous study (Sawabe et al., 2013; Gabriel et al., 2014) were shown and strains belonging to the same clade were labeled with the same color. Among 164 concatenated sequences, 110 of them were obtained by amplification from 98 unknown *Vibrio* strains isolated in this study and 12 standard *Vibrio* strains (See Materials and Methods); while the remaining 54 sequences of reference that have been previously used in MLSA analysis for identification of Species in the Genus *Vibrio* (Gabriel et al., 2014) were retrieved from Genbank.

Supplemental Figure 2 Phylogenetic tree based on the nucleotide sequence of T3SS1 effector coding regions from 62 T3SS1-positive strains. Phylogenetic analysis was conducted as described in Supplemental Figure 1, and the tree was also edited using the Evolview online. Values of bootstrap after 1,000 replications were shown in each node branch represented by the sized circles. Strains belonging to the same species were labeled with the same color.

Supplemental Figure 3 Comparisons of the T3SS1 effector coding region of *V. harveyi* with other *Vibrio* species. The *Vibrio* genomes that contain the genomic loci spanning the T3SS1 effectors were inspected and compared by MultiGeneBLAST. The colors of the size-scaled gene arrows represented BLAST identities across both intra- and inter-specific comparisons. The white gene arrows denote flanking genes without BLAST hits to the query. Syntenic gene tracks showed the co-occurrence of afp17 and its adjacently upstream gene were widely found in several different *Vibrio* species.

Supplemental Table 1 Strains used in this study.

Supplemental Table 2 The primer sequences used in this study.

Supplemental Table 3 GeneBank accession number of nucleotide sequences.

Supplemental Table 4 Sequences used to analyze Afp17 and the results of prediction.
