# Supplementary figures and images for: Type III Secretion 1 Effector Gene Diversity Among Vibrio Isolates From Coastal Areas in China
Source: Front Cell Infect Microbiol. 2020 Jun 18;10:301. doi: 10.3389/fcimb.2020.00301 (PMC7318850; doi:10.3389/fcimb.2020.00301)

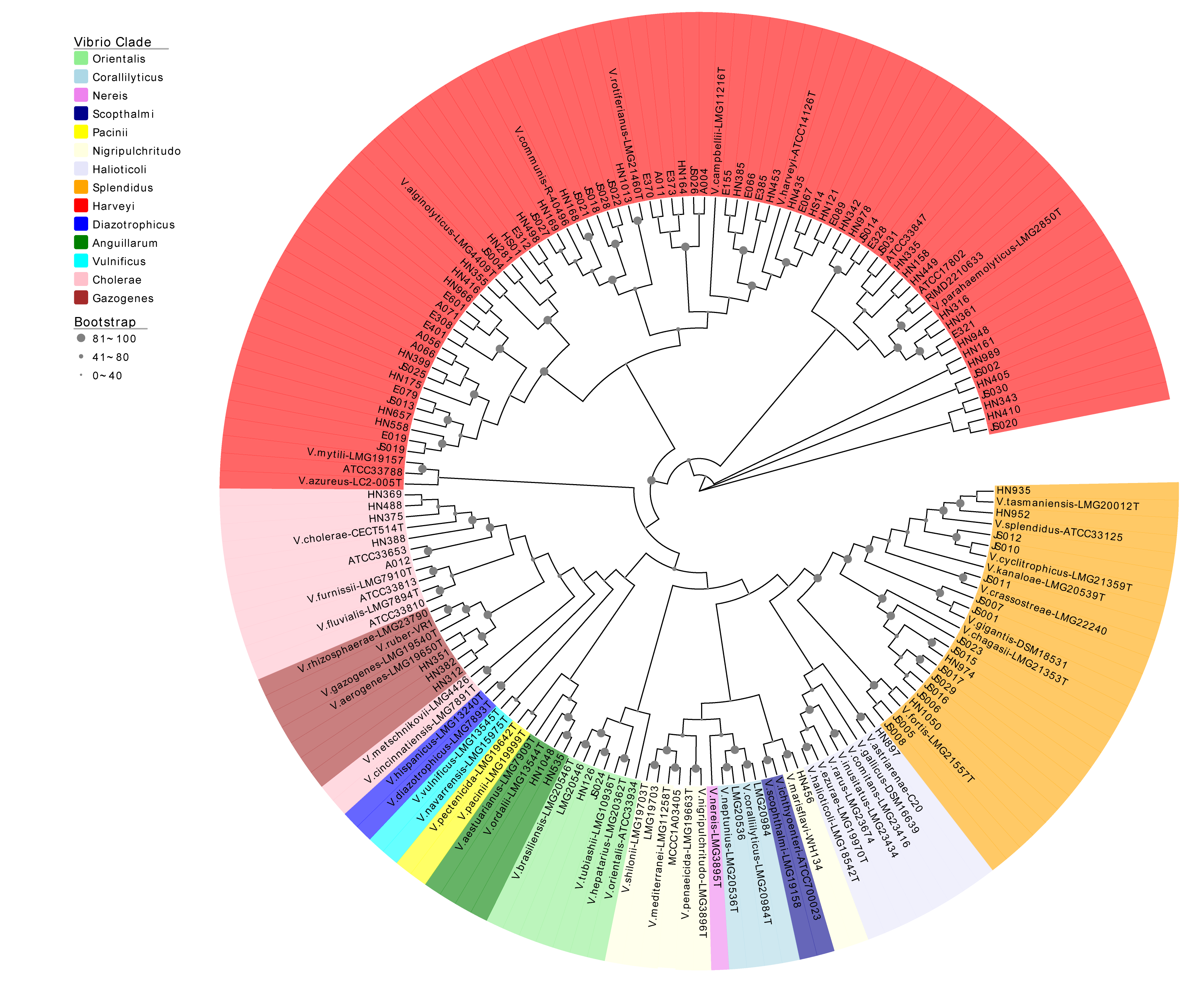

Supplement: Supplemental Figure 1 — Phylogenetic tree of different Vibrio strains. The phylogenetic analysis was based on multiple sequences alignment of 164 concatenated sequences of 16S rRNA gene-gapA-recA-rpoA and conducted by MEGA7 using the Kimura 2-parameter model with the maximum-likelihood method, and then the tree was further edited using the Evolview online. Values of bootstrap after 1,000 replications were shown in each node branch represented by the sized circles. 14 Vibrio clades defined in the previous study (Sawabe et al., 2013; Gabriel et al., 2014) were shown and strains belonging to the same clade were labeled with the same color. Among 164 concatenated sequences, 110 of them were obtained by amplification from 98 unknown Vibrio strains isolated in this study and 12 standard Vibrio strains (See Materials and Methods); while the remaining 54 sequences of reference that have been previously used in MLSA analysis for identification of Species in the Genus Vibrio (Gabriel et al., 2014) were retrieved from Genbank. [file Image_1.TIF]

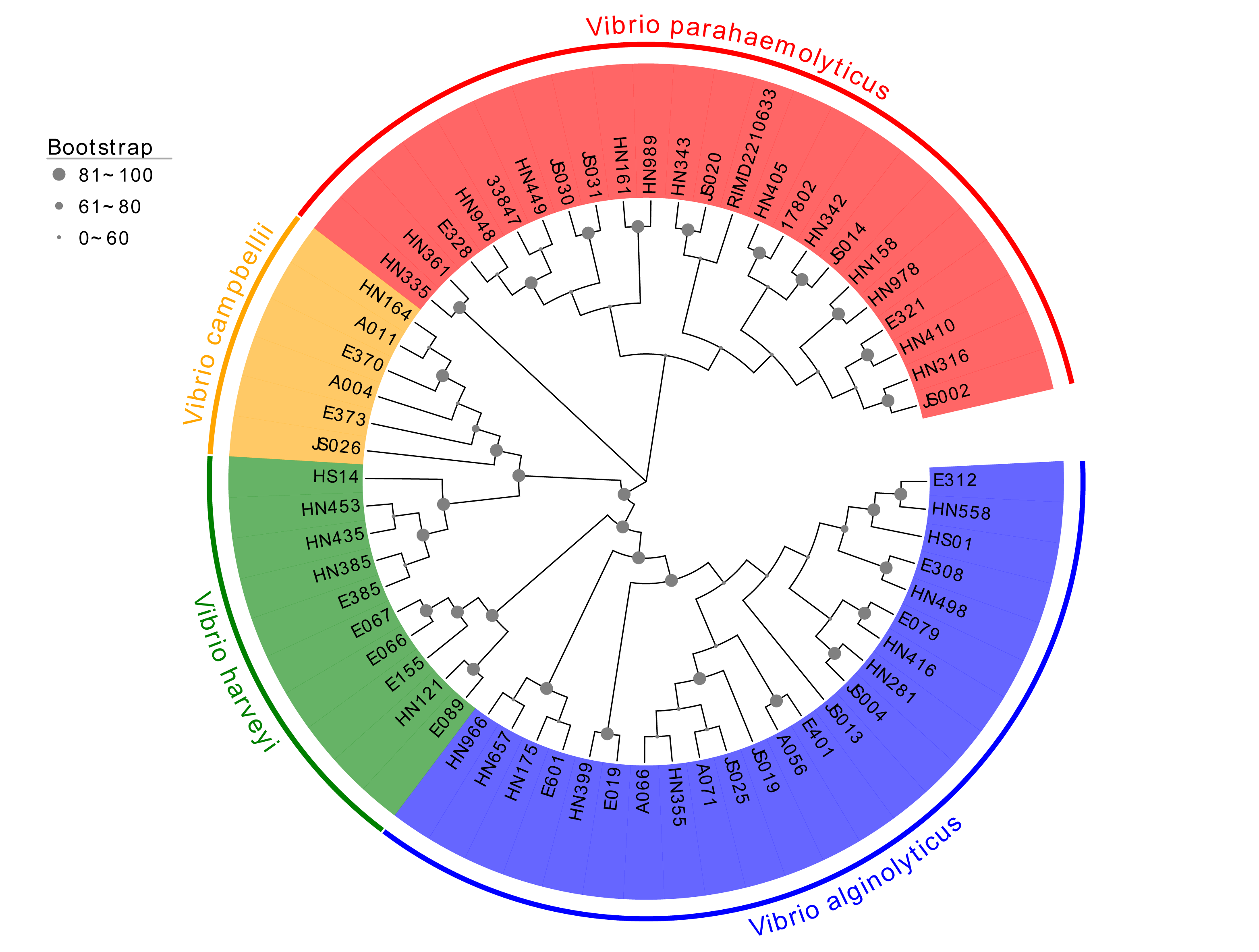

Supplement: Supplemental Figure 2 — Phylogenetic tree based on the nucleotide sequence of T3SS1 effector coding regions from 62 T3SS1-positive strains. Phylogenetic analysis was conducted as described in Supplemental Figure 1, and the tree was also edited using the Evolview online. Values of bootstrap after 1,000 replications were shown in each node branch represented by the sized circles. Strains belonging to the same species were labeled with the same color. [file Image_2.TIF]

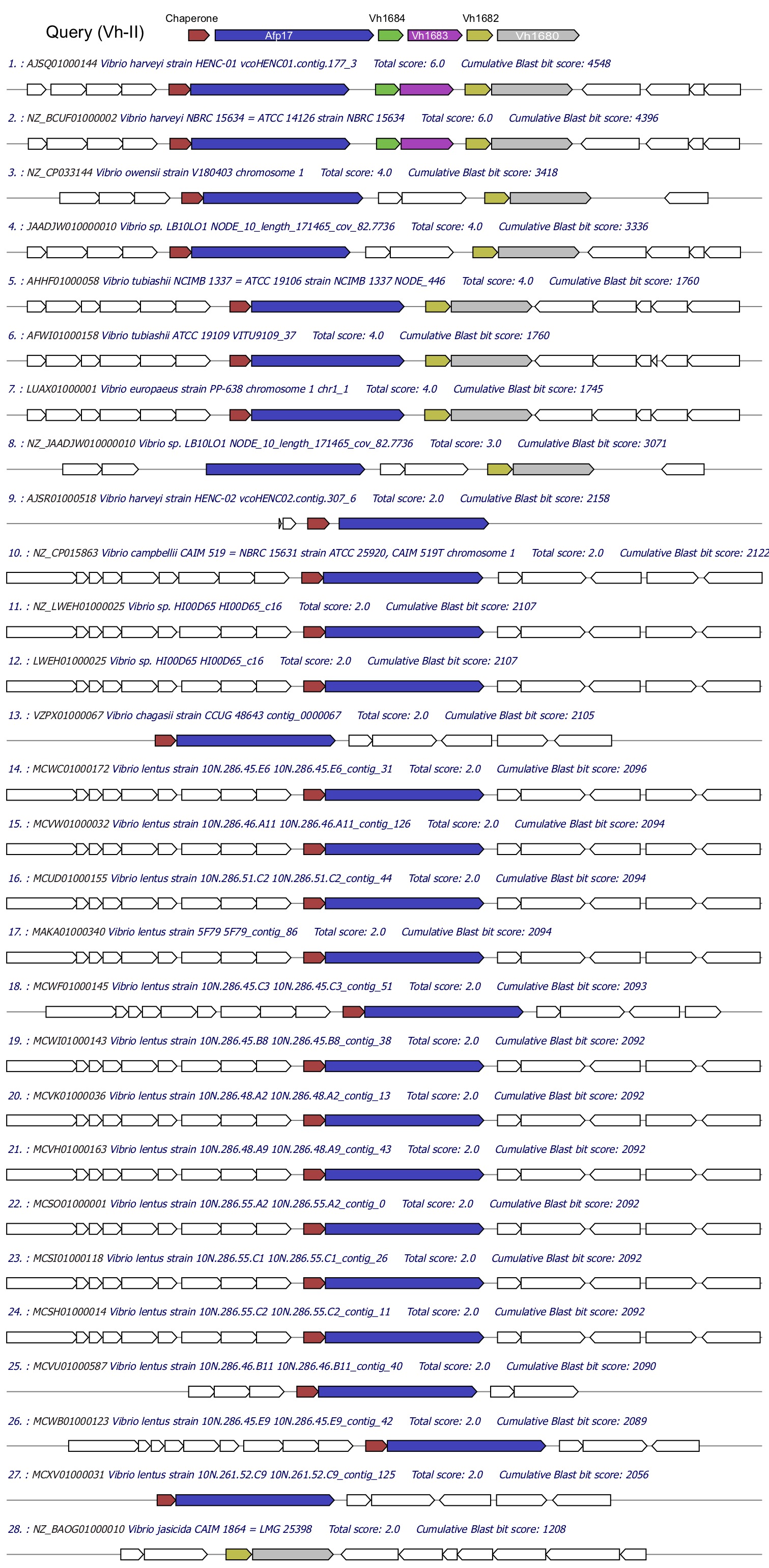

Supplement: Supplemental Figure 3 — Comparisons of the T3SS1 effector coding region of V. harveyi with other Vibrio species. The Vibrio genomes that contain the genomic loci spanning the T3SS1 effectors were inspected and compared by MultiGeneBLAST. The colors of the size-scaled gene arrows represented BLAST identities across both intra- and inter-specific comparisons. The white gene arrows denote flanking genes without BLAST hits to the query. Syntenic gene tracks showed the co-occurrence of afp17 and its adjacently upstream gene were widely found in several different Vibrio species. [file Image_3.JPEG]
